# Repurposing of the Nootropic Drug Vinpocetine as an Analgesic and Anti-Inflammatory Agent: Evidence in a Mouse Model of Superoxide Anion-Triggered Inflammation

**DOI:** 10.1155/2019/6481812

**Published:** 2019-03-31

**Authors:** Yuri Lourenco-Gonzalez, Victor Fattori, Talita P. Domiciano, Ana C. Rossaneis, Sergio M. Borghi, Tiago H. Zaninelli, Catia C. F. Bernardy, Jose C. Alves-Filho, Thiago M. Cunha, Fernando Q. Cunha, Rubia Casagrande, Waldiceu A. Verri

**Affiliations:** ^1^Departamento de Ciências Patológicas, Centro de Ciências Biológicas, Universidade Estadual de Londrina, Rod. Celso Garcia Cid km 480 PR 445, Londrina, Paraná, Brazil; ^2^Departamento de Farmacologia, Faculdade de Medicina de Ribeirão Preto, Universidade de São Paulo, Av. Bandeirantes 3900, Ribeirão Preto, São Paulo, Brazil; ^3^Departmento de Enfermagem, Centro de Ciências da Saúde, Universidade Estadual de Londrina, Av. Robert Koch 60, Londrina, PR, Brazil; ^4^Departamento de Ciências Farmacêuticas, Centro de Ciências da Saúde, Universidade Estadual de Londrina, Av. Robert Koch 60, Londrina, Paraná, Brazil

## Abstract

Clinically active drugs for the treatment of acute pain have their prescription limited due to the significant side effects they induce. An increase in reactive oxygen species (ROS) has been linked to several conditions, including inflammation and pain processing. Therefore, new or repurposed drugs with the ability of reducing ROS-triggered responses are promising candidates for analgesic drugs. Vinpocetine is a clinically used nootropic drug with antioxidant, anti-inflammatory, and analgesic properties. However, the effects of vinpocetine have not been investigated in a model with a direct relationship between ROS, inflammation, and pain. Based on that, we aimed to investigate the effects of vinpocetine in a model of superoxide anion-induced pain and inflammation using potassium superoxide (KO_2_) as a superoxide anion donor to trigger inflammation and pain. In the KO_2_ model, vinpocetine dose-dependently reduced pain-like behaviors (spontaneous pain and hyperalgesia), paw edema, and neutrophil and mononuclear cell recruitment to the paw skin (assessed by H&E staining, fluorescence, and enzymatic assays) and to the peritoneal cavity. Vinpocetine also restored tissue endogenous antioxidant ability and *Nrf2* and *Ho-1* mRNA expression and reduced superoxide anion production and *gp91^phox^* mRNA expression. We also observed the inhibition of I*κ*B*α* degradation by vinpocetine, which demonstrates a reduction in the activation of NF-*κ*B explaining the diminished production of IL-33, IL-1*β*, and TNF-*α*. Collectively, our data show that vinpocetine alleviates pain and inflammation induced by KO_2_, which is a mouse model with a direct role of ROS in triggering pain and other inflammatory phenomena. Thus, the results suggest the repurposing of vinpocetine as an anti-inflammatory and analgesic drug.

## 1. Introduction

Compelling evidence has demonstrated that pain development, at least in part, depends on cellular alterations induced by reactive oxygen species (ROS) [1, 2]. ROS can activate sensory neurons via transient receptor potential cation channel, subfamily A, member 1 (TRPA1, a receptor expressed by sensory neurons), which senses disturbances in ROS metabolism [3, 4]. Focusing on the superoxide anion, it has been reported that extracellular superoxide anion induces neuronal firing, which indicates that it can activate neurons [5]. Furthermore, the injection of ROS donors elicits the spontaneous sensitization of pain and nociceptor sensory neurons that is observed as hyperalgesic responses in rodents [6, 7] and treatment with molecules with antioxidant properties reduces these behaviors [7–10], indicating a role for ROS in pain. Focusing on potassium superoxide (KO_2_), injection of this superoxide anion donor induces a pain phenotype, which is amenable to clinically used drugs, such as morphine (opioid drug) and celecoxib (cyclooxygenase-2 (COX-2) selective inhibitor) [7]. Moreover, *Cox-2* [7] and *Endothelin-1* [11] expression contribute to pain induced by KO_2_. In the case of COX-2, its ultimate product prostaglandin E_2_ (PGE_2_) sensitizes nociceptor neurons and endothelin-1 activates nociceptor neurons [12–14]. The increase of these mediators might be related to the ability of the superoxide anion to activate the nuclear factor-*κ*B (NF-*κ*B) signaling pathway [8, 15, 16]. Thus, the superoxide anion and other ROS produce pain by inducing nociceptor neuron depolarization and by activating pain-related signaling pathways. Leukocyte recruitment and edema development during inflammation are affected by ROS, giving them an important role in inflammatory conditions [2, 6, 8, 15].

Inadequate management of acute pain negatively impacts numerous aspects of patient health and may increase the risk of developing chronic pain [17]. Moreover, if not managed correctly acute pain can cause impaired sleep [17], which is linked to the worsening of pain perception [18]. Therefore, proper care of acute inflammatory pain is very important. Prescription of standard care drugs for acute pain relief relies on the intensity of this pain. Nonsteroidal anti-inflammatory drugs (NSAIDs) are prescribed for mild to moderate pain and with acetaminophen (alone or in combination) for severe pain [19]. However, NSAIDs must be used with caution in conditions such as cardiovascular, renal, or hepatic disease or in patients with risk factors to develop these disease conditions [19]. In fact, diclofenac, a widely used NSAID induces kidney injury and acetaminophen (paracetamol) induces liver injury in mice [20, 21] and humans [22, 23]. Thus, novel therapies with fewer side effects and contraindications showing equivalent efficacy are needed.

Vinpocetine (ethyl apovincamine-22-oate) is a derivative of the molecule vincamine, which is an alkaloid extracted from the leaves of *Vinca minor* [24, 25]. Vinpocetine is a nootropic drug clinically used in the treatment of cognitive impairment. Interestingly, a growing body of evidence has pointed to vinpocetine as a promising candidate due to its anti-inflammatory, antioxidant, and analgesic properties [20, 24–28]. Preclinical data show that vinpocetine does not induce liver or kidney injury in mice [20]. Clinically, patients receiving vinpocetine as a treatment for cerebrovascular diseases and Alzheimer's disease did not show any relevant side effects during treatment [29–31]. Therefore, to date, vinpocetine at therapeutic doses has shown no significant side effects or toxicity and it is considered a safe drug for long-term use [29, 30, 32]. Evidence has demonstrated that vinpocetine reduces oxidative stress and NF-*κ*B activation and thereby induces analgesia in a model of carrageenan- [26] and lipopolysaccharide- (LPS-) induced pain [24]. In terms of abdominal pain, vinpocetine demonstrated an analgesic effect in acetic acid-induced mouse colitis [33] and abdominal contortions [28]. However, the capability of vinpocetine has not been investigated in a model with a direct relationship between ROS, inflammation, and pain. Therefore, the aim of this work is to evaluate the efficacy of vinpocetine in a model of superoxide anion-induced pain and inflammation using KO_2_ as a superoxide anion donor [6, 7].

## 2. Materials and Methods

### 2.1. Animals

The present study used male Swiss mice or LysM-eGFP C57BL/6 background mice from Londrina State University, Paraná, Brazil. The weight of the mice selected for the study was between 20 and 25 g. The mice were separated in appropriate plastic cages according to their respective groups, with food and water *ad libitum* and a light/dark-programmed 12/12 h cycle in a room with a temperature of 21 ± 1°C and an air exhaust. Behavioral analyses were always conducted during the light cycle. Euthanasia was performed by three sequential procedures to minimize animal suffering. First, mice were anesthetized with a sublethal dose (5%) of isoflurane, followed by cervical dislocation, and subsequently decapitation. All experiments, including handling procedures and animal care, were approved under process number 6166.2014.85 by the Animal Ethics Committee of the Universidade Estadual de Londrina and followed the guidelines of the International Association for the Study of Pain (IASP). To minimize the number of animals used, all experiments were accurately programmed. Accidental or sudden animal deaths did not occur during the development of the study.

### 2.2. Drugs and Reagents

Drugs and reagents used in the present study were obtained from the following sources: potassium superoxide (KO_2_) 96.5% was from Alfa Aesar (Ward Hill, MA, USA); vinpocetine powder at >98% purity was from Santa Cruz Biotechnology (Dallas, TX, USA); saline solution (NaCl 0.9%) was from Frenesius Kabi Brasil Ltda (Aquiraz, CE, Brazil); isoflurane was from Abbott Laboratories (Abbott Park, IL, USA); nitroblue tetrazolium (NBT) was from Amresco (Solon, OH, USA); and ferric chloride hexahydrate, 2,4,6-tripyridyl-s-triazine (TPTZ) and ABTS (2,20-azino-bis(3-ethylbenzothiazoline-6-sulfonate)) was from Sigma-Aldrich (St. Louis, MO, USA).

### 2.3. General Experimental Procedures

One hour before intraplantar (i.pl.) stimulus of vehicle (control group, saline, 25 *μ*L) or KO_2_ (30 *μ*g, 25 *μ*L), mice were treated per oral (p.o.) with vehicle (sterile saline, 100 *μ*L) or vinpocetine (3, 10, or 30 mg/kg, diluted in sterile saline). Abdominal contortions were the first experiment performed and used to determine the best dose of vinpocetine for subsequent experiments involving overt pain-like behavior (number of flinches and time spent licking the paw). Focusing on hyperalgesia, the optimal dose of vinpocetine (30 mg/kg) was chosen based on mechanical and thermal hyperalgesia assays. After that, the following additional experiments were performed: neutrophil and macrophage recruitment to the paw skin 7 h after KO_2_ stimulus (H&E staining, MPO and NAG activities, and LysM-eGFP fluorescence measurement) and neutrophil and macrophage recruitment to the peritoneal cavity 6 h after KO_2_ stimulus (total leukocytes, mononuclear cells, and neutrophils). Oxidative stress assays were performed 3 h after KO_2_ stimulus (reflected by the measurements of antioxidant defenses, superoxide anion production, and mRNA expression of nicotinamide adenine dinucleotide phosphate (NADPH) oxidase subunit *gp91^phox^*, antioxidant responsive elements of (ARE) nuclear factor (erythroid-derived 2)-like 2 (*Nrf2*), and heme-oxygenase-1 (*Ho-*1)). Also, the quantification of the mRNA expression of *Endothelin-1* and *Cox-2*, the production of cytokine (IL-33, TNF*α*, and IL-1*β*), and the activation of NF-*κ*B (I*κ*B*α* degradation) were determined in paw tissue samples 3 h after KO_2_ stimulus was performed. The time point selected for tissue dissection after the injection of KO_2_ as well the dose for both stimulus and vinpocetine were based on previous studies of our laboratory [7, 24, 26].

### 2.4. Abdominal Contortions, Number of Paw Flinches, and Time Spent Licking the Paw

Animal abdominal writhings were induced by intraperitoneal (i.p.) administration of KO_2_ (1 mg) [7]. Right after KO_2_ injections, mice were gently and individually placed in a glass cylinder with enough space for free movement. The total number of abdominal writhing responses in a period of 20 min following KO_2_ i.p. stimulus was quantitated as a measure of nociceptive behavior. For this assay, a positive response was considered when the animal performed a stretching of the hind limbs associated with a slow abdominal wall contraction. Regarding paw flinches and the time spent licking the paw, behaviors were analyzed during 30 min immediately after i.pl. stimulus with KO_2_ (30 *μ*g) [7]. The same conditions and glass utensil described for the use of the abdominal contortion analysis were used for the quantification of paw flinches and the time spent licking the paw. Results were expressed as the total number of paw flinches and the time (seconds) spent licking the KO_2_ stimulated paw.

### 2.5. Mechanical Hyperalgesia

An electronic version of von Frey's filaments (cat #EFF 301, Insight, Ribeirão Preto, SP, Brazil) was used to determine mechanical hyperalgesia, as reported previously [34]. For that, mice were gently placed in acrylic cages (cat #EFF 303, Insight, Ribeirão Preto, SP, Brazil), located in a quiet, temperature-controlled room. Mice were habituated at least 30 min before the start of testing. During the measurements, an experimenter blinded to the treatment exerts pressure on the plantar surface of the animals of different groups using a force transducer coupled with a polypropylene tip (0.5 mm^2^) that results in hind paw flexion reflex. Mechanical stimulation of the plantar hind paw was performed only when animals are quiet and resting over the four paws. A clear flinching movement of the paw can be observed, and the stimulation intensity necessary to trigger this response decreases upon hyperalgesia increase. The equipment is designed to record the pressure intensity upon paw withdrawal. The final response value was an average of three measurements. The animals were tested before (baseline values) and after the treatment. The results are expressed in grams by delta (Δ) withdrawal threshold, in which the final values of the indicated time points after the KO_2_ stimulus were subtracted from the baseline measurements [7].

### 2.6. Thermal Hyperalgesia

A hot plate apparatus (cat #EFF 361, Insight, Ribeirão Preto, SP, Brazil) with a temperature of 55°C ± 1°C was used to determine heat thermal hyperalgesia, as described previously [7]. Jumping, clear paw flinching, or paw licking behaviors were considered positive withdrawal responses. The results are expressed by delta (Δ) withdrawal threshold (in seconds), in which the final values of the indicated time points after the KO_2_ stimulus were subtracted from the baseline measurements. Care was taken to avoid any potential tissue damage by setting the cutoff at 20 sec [9]. The experimenters were always blinded to the groups.

### 2.7. Paw Edema Measurement

At indicated time points, paw edema was measured using a conventional caliper (Digimatic Caliper, Mitutoyo Corporation, Kanagawa, Japan). Results are expressed in millimeters as the difference between the paw thickness measured before (baseline values) and at the indicated time points after the KO_2_ stimulus [7]. The investigators were blinded to the groups.

### 2.8. Paw Tissue Histology

After dissection (7 h after KO_2_ stimulus), paraffin-embedded hind paw tissue was processed for hematoxylin and eosin (H&E) staining. Samples were fixed with 10% paraformaldehyde in PBS prior to embedding. Digitally acquired images were analyzed and scored in a conventional light microscope (40x objective) by an experimenter blinded to the treatment. Analyses were performed on ImageJ 1.44 software for Windows (Java image software in public domain: http://rsb.info.nih.gov/ij/) using the threshold tool and performed on RGB images without further treatment. Leukocyte recruitment was determined using 1086333 pixels as the dimension area.

### 2.9. Myeloperoxidase (MPO) and N-Acetyl-*β*-D-01.12.0294.00 (0476/11)glucosaminidase (NAG) Assays

The MPO assay was used to evaluate the mobilization of neutrophils to the paw tissue in response to KO_2_ stimulus as described previously [35]. Ten *μ*L of the resultant supernatant was incubated with 200 *μ*L of 50 mM phosphate buffer, pH 6.0, containing 0.167 mg/mL *o*-dianisidine dihydrochloride and 0.015% hydrogen peroxide. Reading was performed at 450 nm (Multiskan GO microplate spectrophotometer, Thermo Fisher Scientific, Vantaa, Finland) and the MPO activity was compared to a standard curve of neutrophils with the results expressed as the MPO activity (number of neutrophils × 10^4^/mg of skin paw). For the NAG activity, 20 *μ*L of supernatant was obtained as described for the MPO activity assay and added to a 96-well plate, followed by the addition of 80 *μ*L of 50 mM phosphate buffer, pH 6.0 [16]. The plate was incubated with 2.24 mM 4-nitrophenyl N-acetyl-*β*-D-glucosaminide (37°C, 10 min). Reading was performed at 400 nm (Multiskan GO microplate spectrophotometer, Thermo Fisher Scientific, Vantaa, Finland) after the reaction was stopped by the addition of 100 *μ*L of 0.2 M glycine buffer, pH 10.6. The NAG activity of the samples was compared to a standard curve of macrophages and presented as the NAG activity (number of macrophages × 10^3^/mg of skin paw).

### 2.10. Paw Tissue Fluorescence

Optimum cutting temperature reagent- (Tissue-Tek 1, O.C.T. Compound, IA018, ProSciTech, Australia) embedded paw tissue dissected from LysM-eGFP^+^ C57BL/6 background mice were used for this assay. LysM-eGFP mice express enhanced green fluorescent protein (eGFP) expression controlled by the lysozyme M promoter (LysM) present in neutrophils and macrophage granules. Hind paw tissue was dissected 7 h after i.pl. stimulus with KO_2_ and maintained in 4% paraformaldehyde (PFA, 24 h) and then in 30% sucrose (72 h). Fluoromount-G reagent (00-4958-02, Thermo Fisher Scientific, Waltham, MA, USA) was added to 20 *μ*m sections that were put in slides to complete their assembly. Imaging was performed using a confocal microscope (Leica TCS SP8, Leica, Wetzlar, Germany) with a 40x objective. Images were processed using Leica EL6000 software (Leica, Wetzlar, Germany). The intensity of fluorescence was quantified by an investigator blinded to the treatment in randomly selected fields (one field per sample, *n* = 4) of different groups as an indication of neutrophil/macrophage recruitment to the paw tissue. The results are expressed as eGFP fluorescence intensity (%).

### 2.11. Leukocyte Recruitment to the Peritoneal Cavity

Leukocyte migration to the peritoneal cavity was evaluated in a light microscope (400x magnification, Olympus Optical Co., Hamburg, Germany) 6 h after i.p. injection of KO_2_ (30 *μ*g/cavity) using peritoneal wash [7]. Total leukocyte counts were carried out using a Neubauer chamber in Turk's solution (2% acetic acid). Differential cell counts (mononuclear cells and neutrophils) were determined through staining with a Fast Panoptic Commercial Kit (Laborclin, Pinhais, PR, Brasil). Results are expressed as the total number of cells (×10^6^) per peritoneal cavity.

### 2.12. Superoxide Anion Production: NBT Assay

Superoxide anion production in paw tissue samples was evaluated using the NBT reduction assay, as previously described [9]. Samples were incubated (37°C, 1 h) with an NBT reagent (100 *μ*L, 1 mg/mL). The supernatant was then accurately removed by pipetting, and the reduced formazan formed was solubilized with KOH (120 *μ*L) and DMSO (140 *μ*L). Measurement was performed immediately after adding KOH and DMSO at 600 nm using a microplate spectrophotometer reader (Multiskan GO, Thermo Fisher Scientific). The results are expressed as NBT reduction (OD/mg of paw skin tissue).

### 2.13. Total Antioxidant Capacity: Ferric Reducing Ability Potential (FRAP) and Ability to Scavenge the 2′2′-Azino-bis(3-ethylbenzothiazoline-6-sulphonic Acid (ABTS) Radical Assays

The capacity to counteract oxidative deleterious effects was evaluated in paw skin samples by FRAP and ABTS tests 3 h after the KO_2_ stimulus [11]. Samples of paw skin tissue were dissected and homogenized in KCl buffer, for subsequent centrifugation (200 g × 10 min × 4°C). The supernatant was used for both FRAP and ABTS tests. For the FRAP assay, samples were incubated (37°C, 30 min) with 150 *μ*L of FRAP reagent and read at 595 nm (Multiskan GO, Thermo Fisher Scientific, Vantaa, Finland), while for the ABTS assay, samples were incubated (25°C, 6 min) with 200 *μ*L of ABTS and read at 730 nm. A standard Trolox curve (0.02–20 nmol) was used to equalize FRAP and ABTS tests. The results are presented as nanomols of Trolox equivalent/milligram of paw tissue.

### 2.14. Cytokine Measurement

Paw skin tissue was dissected into 500 *μ*L of ice-cold buffer containing protease inhibitors. After this step, samples were centrifuged (3000 rpm × 10 min × 4°C), and the resultant supernatants were used to determine IL-33, TNF-*α*, and IL-1*β* concentrations by enzyme-linked immunosorbent assay (ELISA), using eBioscience commercial kits. For the test, 96-well plates were initially coated with specific antibodies for each cytokine of interest and then blocked with recombinant murine standards for each cytokine. In the next phases, incubations with antibodies against each cytokine and avidin-HRP were carried out following the manufacturer's instructions. The levels of the evaluated cytokines were also determined in animals that received saline solution as a control. The measurements were conducted at 450 nm. The results are expressed as picograms (pg) of cytokine/mg of paw tissue.

### 2.15. Reverse Transcription and Quantitative Polymerase Chain Reaction (RT-qPCR)

Samples were dissected into the TRIzol® reagent for RNA isolation, which was performed according to the manufacturer's guidelines. All total RNA used in the reactions for cDNA presented 1.8 and 2.0 as the OD ratio measured at 260/280 nm. RT-qPCR was performed using the GoTaq® 2-Step RT-qPCR System (Promega) and specific primers (Applied Biosystems®). The primer sequences used in this work are as follows: *β-actin*, sense: 5′-AGCTGCGTTTTACACCCTTT-3′, antisense: 5′-AAGCCATGCCAATGTTGTCT-3′; *gp91^phox^*, sense: 5′-AGCTATGAGGTGGTGATGTTAGTGG-3′, antisense: 5′-CACAATATTTGTACCAGACAGACTTGAG-3′; *Nrf2*, sense: 5′-TCACACGAGATGAGCTTAGGGCAA-3′, antisense: 5′-TACAGTTCTGGGCGGCGACTTTAT-3′; Heme Oxygenase-1 (*Ho-1*), sense: 5′-CCCAAAACTGGCCTGTAAAA-3′, antisense: 5′-CGTGGTCAGTCAACATGGAT-3′; *Endothelin-1*: sense: 5′-TGTGTCTACTTCTGCCACCT-3′, antisense: 5′-CACCAGCTGCTGATAGATAC-3′; Cycloxygenase-2 (*Cox-2*): sense: 5′-GTGGAAAAACCTCGTCCAGA-3′, antisense: 5′-GCTCGGCTTCCAGTATTGAG-3′. The relative gene expression was measured using the comparative 2^-(∆∆Ct)^ method using *β-actin* as a reference gene to normalize data.

### 2.16. Western Blot Assay

Samples were dissected 3 h after the injection of KO_2_. Western blotting was performed as described previously [16] using primary antibodies anti-I*κ*B*α* (#9242, 1 : 1000) or anti-*β*-actin (#4970, 1 : 1000) as a loading control (Cell Signaling Technology, Santa Cruz, CA, USA). ImageJ software (NIH, Bethesda, MD, USA) was used to measure the optical density of the bands.

### 2.17. Statistical Analysis

All data were analyzed using the software GraphPad Prism 6.01. Experiments were conducted twice (independent experiments) using six mice in each group per experiment, except for those involving LysM-eGFP in which were used four mice in each group per experiment. Results are presented as means ± SEM of those measurements. Two-way repeated measure analysis of variance (ANOVA) followed by Tukey's post hoc was used for thermal hyperalgesia, mechanical hyperalgesia, and paw edema (measurements in different time points after the stimulus injection). Other experiments were analyzed using one-way ANOVA followed by Tukey's *post hoc* (measurement in a single time point after the stimulus injection). Statistical differences were considered significant when *P* < 0.05.

## 3. Results

### 3.1. Vinpocetine Decreases Writhing, Paw Flinches, and Time Spent Licking the Paw Induced by Superoxide Anion

**Figure 1:**
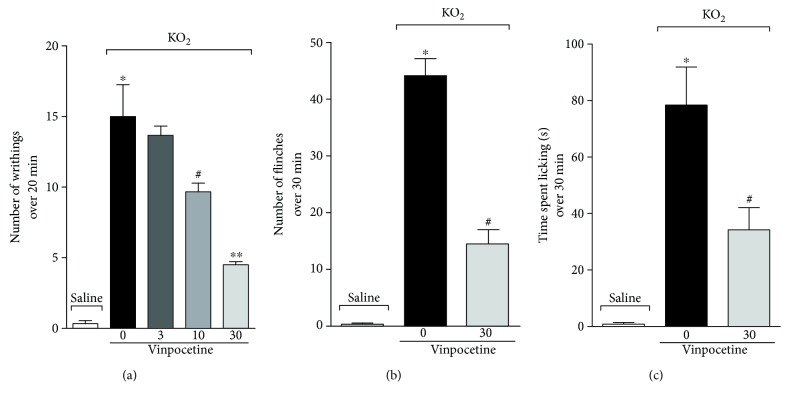
Vinpocetine decreases writhing, flinching of the paw, and time spent licking the paw induced by superoxide anion. The number of abdominal contortions over 20 min (a) after intraperitoneal injection of KO_2_ (1 mg/cavity). The number of paw flinches (b) and time spent licking the paw (c) were determined over 30 min after intraplantar injection of KO_2_ (30 *μ*g/paw) (^∗^*p* < 0.05 vs. saline group; ^#^*p* < 0.05 vs. vehicle (0 mg/kg) group; ^∗∗^*p* < 0.05 vs. 10 mg/kg, one-way ANOVA followed by Tukey's posttest; mean ± SEM).

First, a dose-response curve was used to determine the dose of vinpocetine capable of reducing spontaneous pain behaviors. Vinpocetine at doses of 10 and 30 mg/kg reduced KO_2_-induced abdominal writhing (Figure 1(a)) by approximately 38% and 70%, respectively (*F* (4, 25) = 32.31, *p* < 0.0001) with an ID_50_ of 15.32 mg/kg (95% confidence interval: ID_50_ 12.32 to 19.05 mg/kg). Thirty mg/kg was used for the following experiments involving overt pain-like behaviors given that its analgesic effect was statistically different when compared to the dose of 10 mg/kg (*p* < 0.01) in the writhing test. Vinpocetine at 30 mg/kg also reduced the amount of paw flinches (Figure 1(b)) and the time (seconds) which the mouse spent licking the paw injected with KO_2_ (Figure 1(c)).

### 3.2. KO_2_-Induced Hyperalgesia (Mechanical and Thermal) and Paw Edema Are Reduced by Vinpocetine

**Figure 2:**
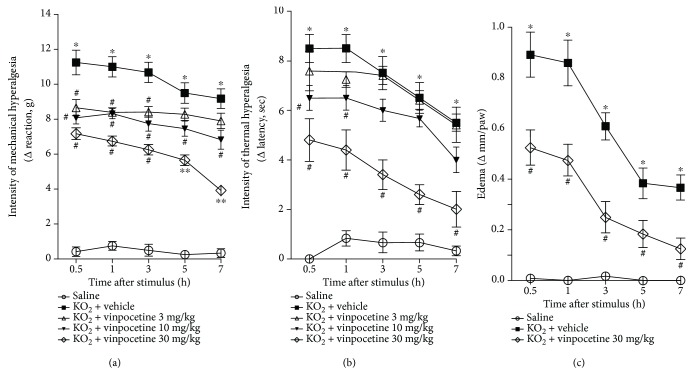
KO_2_-induced hyperalgesia (mechanical and thermal) and paw edema are reduced by vinpocetine. Mechanical hyperalgesia (a), thermal hyperalgesia (b), and paw edema (c) were evaluated 0.5, 1, 3, 5, and 7 h after intraplantar injection of KO_2_ (30 *μ*g/paw). Mechanical hyperalgesia is presented as Δ withdrawal threshold (in grams), and thermal hyperalgesia is presented as Δ withdrawal threshold (in seconds), both of which were calculated by subtracting the mean measurements at 0.5, 1, 3, 5, and 7 h after KO_2_ stimulus from the baseline mean measurements (before stimulus) (^∗^*p* < 0.05 vs. saline, ^#^*p* < 0.05 vs. vehicle (0 mg/kg) group, ^∗∗^*p* < 0.05 vs 10 mg/kg; two-way repeated measures ANOVA followed by Tukey's posttest; Δ mean ± SEM).

Next, it was investigated whether vinpocetine could reduce KO_2_-induced mechanical hyperalgesia, thermal hyperalgesia, and paw edema. The results are presented as the delta value from the baseline (before stimulus); thus, a higher the delta value means increased hyperalgesia. Mechanical hyperalgesia was reduced at all time points upon treatment with vinpocetine (10 and 30 mg/kg) (Figure 2(a)). The dose of 30 mg/kg presented statistical difference when compared to the dose of 10 mg/kg at 5 and 7 h after stimulus with KO_2_. Only vinpocetine at 30 mg/kg decreased thermal hyperalgesia at all time points (Figure 2(b)). Thus, the dose of 30 mg/kg was chosen for the following experiments. Vinpocetine at 30 mg/kg was able to reduce paw edema (Figure 2(c)).

### 3.3. Vinpocetine Inhibits the Recruitment of Neutrophils and Macrophages to the Paw Skin after Stimulus with KO_2_

**Figure 3:**
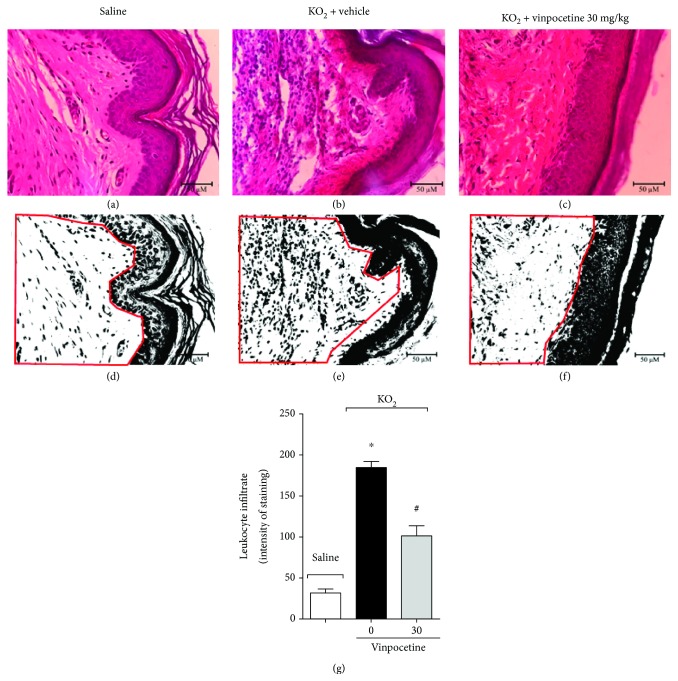
Vinpocetine inhibits superoxide anion-induced leukocyte recruitment to the paw skin. Seven hours after intraplantar injection of KO_2_ (30 *μ*g/paw), the hind paw skin was dissected for histopathological analysis by H&E staining using a light microscope (original magnification 40x). The representative total score of leukocyte recruitment is presented in (g) and determined using ImageJ software in 1086333 pixels of dimension area (highlighted area in red (d-f)) (^∗^*p* < 0.05 vs. saline group; ^#^*p* < 0.05 vs. vehicle (0 mg/kg) group, one-way ANOVA followed by Tukey's posttest; mean ± SEM).

**Figure 4:**
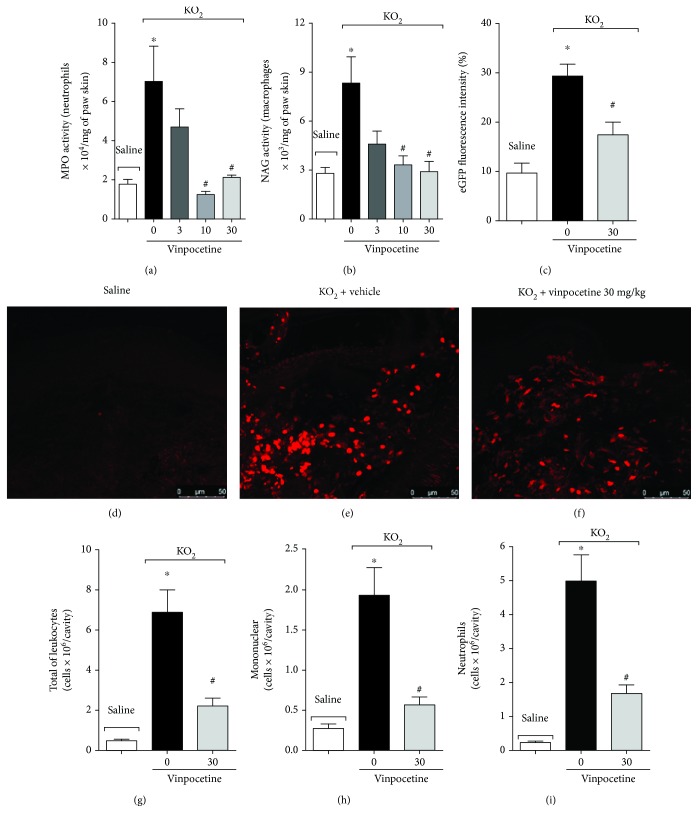
Vinpocetine inhibits the recruitment of neutrophils and macrophages to the paw skin after KO_2_ stimulus. Seven hours after intraplantar injection of KO_2_ (30 *μ*g/paw), the hind paw skin of Swiss mice was dissected to determine MPO ((a) neutrophil marker) and NAG ((b) macrophage marker) activities. In experiments involving LysM-eGFP mice, the hind paw skin was dissected seven hours after intraplantar injection of KO_2_ (30 *μ*g/paw) for the determination of fluorescence intensity using a confocal microscope. Percentage of fluorescence is represented in (c) and representative images are shown in (d)-(f). Six hours after intraperitoneal injection of KO_2_ (30 *μ*g/cavity), the peritoneal wash was collected to determine the recruitment of total leukocytes (g), mononuclear cells (h), and neutrophils (i) (^∗^*p* < 0.05 vs. saline group; ^#^*p* < 0.05 vs. vehicle (0 mg/kg) group, one-way ANOVA followed by Tukey's posttest; mean ± SEM).

Immune cells, such as neutrophils and macrophages, are recruited upon noxious stimuli and play an important role in the generation and maintenance of pain [36, 37]. Given that, we next investigated the effect of vinpocetine on this parameter. We first performed histopathological analysis focusing on the dermal region of the hind paw skin using H&E staining. Our result shows that treatment with vinpocetine at 30 mg/kg reduced total leukocyte infiltration (Figures 3(a)–3(g)). To have a better readout of neutrophil and macrophage recruitment, enzymatic assays were also performed. Treatment with vinpocetine at doses of 10 and 30 mg/kg reduced neutrophil (MPO activity, Figure 4(a)) and macrophage (NAG activity, Figure 4(b)) recruitment. Since H&E staining does not give a precise measurement of leukocyte recruitment to the paw skin, LysM-eGFP mice were used as a further approach to investigate this parameter. Treatment with vinpocetine reduced the infiltration of LysM-eGFP+ cells as observed by a reduced percentage of fluorescence using a confocal microscope (Figure 4(c)) and representative images (Figures 4(d)–4(f)). After intraperitoneal injection, KO_2_ induces the recruitment of leukocytes to the peritoneal cavity [7]. We next evaluated whether vinpocetine could reduce this parameter. Treatment with vinpocetine at the dose of 30 mg/kg diminished the recruitment of total leukocytes (Figure 4(g)), neutrophils (Figure 4(h)), and mononuclear cells (Figure 4(i)) upon KO_2_ i.p. stimulus.

### 3.4. Vinpocetine Normalizes Total Antioxidant Capacity and Reduces Superoxide Anion Production Induced by KO_2_

**Figure 5:**
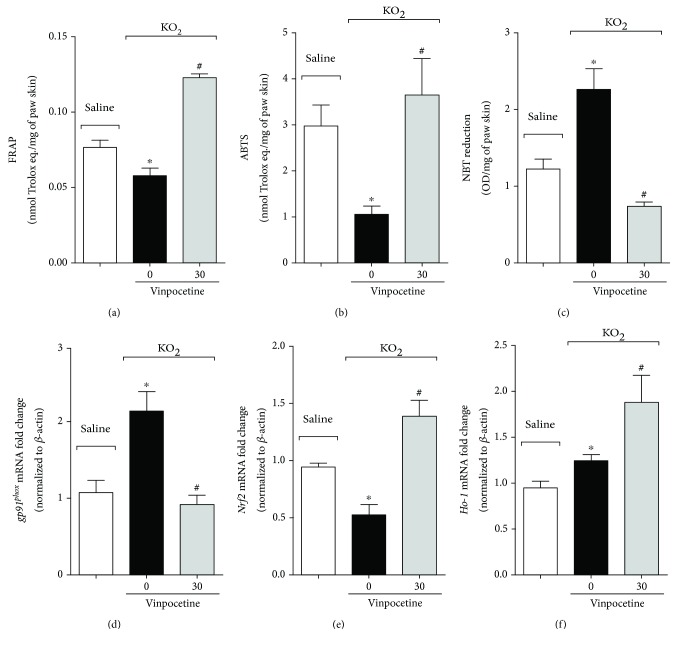
Vinpocetine normalizes total antioxidant capacity and reduces superoxide anion production induced by KO_2_. Total antioxidant capacity was quantified using FRAP (a) and ABTS (b) assays. Production of superoxide anion was determined by NBT assay (c). RT-qPCR was used to determine the *gp91^phox^* (d), *Nrf2* (e), and *Ho-1* (f) mRNA expression (^∗^*p* < 0.05 vs. saline group; ^#^*p* < 0.05 vs. vehicle (0 mg/kg) group, one-way ANOVA followed by Tukey's posttest; mean ± SEM).

Given that this is a model of pain with a direct relationship with ROS [6, 7], we assessed the capacity of vinpocetine to inhibit KO_2_-induced oxidative stress. Treatment with vinpocetine restored the total antioxidant capacity as observed by the normalization of the capacity to reduce ferric ion (FRAP assay, Figure 5(a)) and scavenger ability (ABTS assay) (Figure 5(b)). Injection of potassium superoxide increases both O_2_^−^ and the NADPH oxidase subunit mRNA expression, *gp91^phox^* [7, 9, 11]. Treatment with vinpocetine reduced superoxide anion production (Figure 5(c)) and decreased *gp91^phox^* mRNA expression (Figure 5(d)). Vinpocetine also normalized the expression of the antioxidant transcription factor *Nrf2* (Figure 5(e)) and its downstream target *Ho-1* (Figure 5(f)) when compared to the vehicle group.

### 3.5. Vinpocetine Reduces Endothelin-1 and Cox-2 mRNA Expression Induced by KO_2_

**Figure 6:**
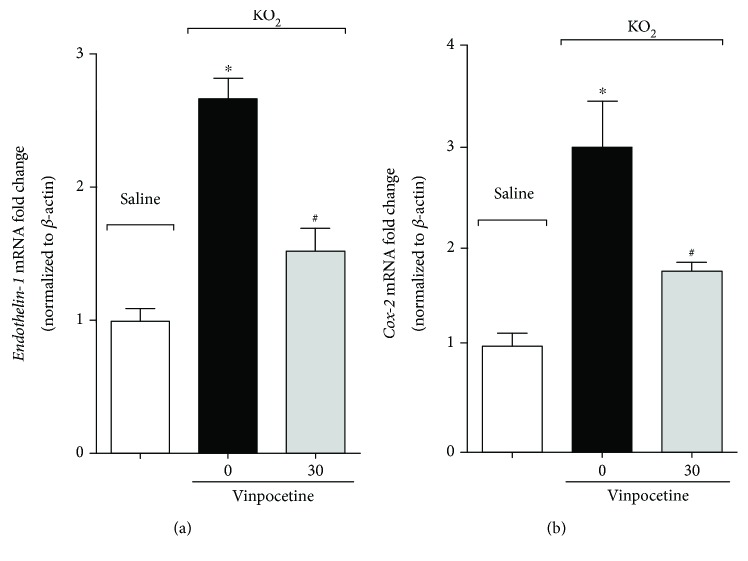
Vinpocetine reduces *Endothelin-1* and *Cox-2* mRNA expression induced by KO_2_. RT-qPCR was used to determine *Endothelin-1* (a) and *Cox-2* (b) mRNA expression. Hind paw skin was dissected three hours after intraplantar injection of KO_2_ (30 *μ*g/paw) (^∗^*p* < 0.05 vs. saline group; ^#^*p* < 0.05 vs. vehicle (0 mg/kg) group, one-way ANOVA followed by Tukey's posttest; mean ± SEM).

NSAIDs have been extensively used as analgesics and act by inhibiting the activity of COX-1 or COX-2, thereby, reducing PGE_2_ synthesis [12, 38, 39]. Given that PGE_2_ sensitizes nociceptor neurons [12, 38] and endothelin-1 activates nociceptor neurons [13, 14], the effect of vinpocetine was evaluated on *endothelin-1* and *Cox-2* mRNA expression in the KO_2_ model. Treatment with vinpocetine diminished the induction of *endothelin-1* (Figure 6(a)) and *Cox-2* (Figure 6(b)) mRNA expression in the KO_2_ model.

### 3.6. Vinpocetine Decreases Superoxide Anion-Induced IL-33, TNF-*α*, and IL-1*β* Production and NF-*κ*B Activation

**Figure 7:**
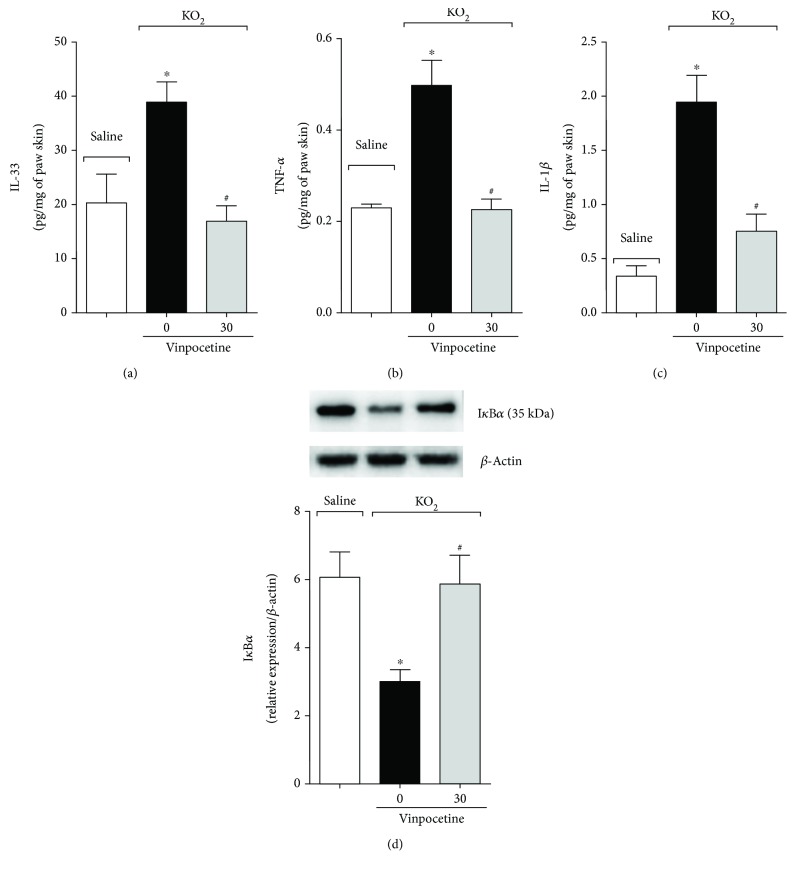
Vinpocetine decreases superoxide anion-induced IL-33, TNF-*α*, and IL-1*β* production and NF-*κ*B activation. The levels of the proinflammatory cytokines IL-33 (a), TNF-*α* (b), and IL-1*β* (c) were assayed by ELISA. The activation of NF-*κ*B (d) was assessed by western blot by measuring the protein levels of I*κ*B*α* (NF-*κ*B inhibitor). Hind paw skin was dissected three hours after intraplantar injection of KO_2_ (30 *μ*g/paw) (^∗^*p* < 0.05 vs. saline group; ^#^*p* < 0.05 vs. vehicle (0 mg/kg) group, one-way ANOVA followed by Tukey's posttest; mean ± SEM).

Strategies targeting cytokines or their receptors are recognized as effective analgesic approaches [40]. Thus, we next investigated the effect of vinpocetine on IL-33, TNF-*α*, and IL-1*β* production and NF-*κ*B activation. Treatment with vinpocetine decreased the KO_2_-triggered production of IL-33 (Figure 7(a)), TNF-*α* (Figure 7(b)), and IL-1*β* (Figure 7(c)). As these cytokines are produced in an NF-*κ*B-dependent manner, we next investigated the effect of vinpocetine over NF-*κ*B activation. We observed an increase in the protein levels of I*κ*B*α* (NF-*κ*B inhibitor) after treatment with vinpocetine, indicating that this molecule reduced KO_2_-induced NF-*κ*B activation (Figure 7(d)).

## 4. Discussion

In this work, we show that vinpocetine reduced inflammation and pain in a model with a direct relationship between pain and ROS. Vinpocetine reduced mechanical and thermal hyperalgesia and spontaneous behaviors, which are considered to be of nociceptive nature. Treatment with vinpocetine restored tissue endogenous antioxidant defenses and *Nrf2/Ho-1* mRNA to baseline levels. We also observed a reduction of superoxide anion production and *gp91^phox^* mRNA expression. Treatment with vinpocetine reduced paw skin levels of the cytokines IL-33, TNF-*α*, and IL-1*β* through the inhibition of NF-*κ*B activation.

Oxidative stress has been shown as an essential factor to the genesis of acute and chronic pain by inducing peripheral and central sensitization [1]. In fact, increased excitability is observed in nociceptors of the dorsal horn of the spinal cord after stimulation with ROS donors [41]. In the periphery, TRPA1-expressing sensory neurons, a receptor that senses disturbances in ROS metabolism, can be activated by ROS [3, 4]. In terms of nociceptive behavioral changes, the injection of ROS induces both hyperalgesia and spontaneous pain in naïve animals [6, 9, 35, 42]. Focusing on the superoxide anion, evidence demonstrated that it can influx through anionic channels causing neuronal firing in medullary dorsal horn neurons [5]. In this sense, molecules with the ability of targeting ROS metabolism are interesting analgesic approaches [1]. The increase of ROS can be reduced directly or indirectly. Molecules with indirect mechanisms act through the activation of antioxidant transcription factors, e.g., Nrf2 [43, 44]. Herein, KO_2_ was used as a superoxide anion donor given that this molecule produces ROS-dependent pain. For example, the alleviation of pain and inflammation can be achieved after treatment with antioxidant molecules such as quercetin [7], curcumin [9], tempol (a mimetic of the superoxide dismutase (SOD) enzyme) [45], or apocynin (an inhibitor of the NADPH oxidase enzyme) [46]. In the present study, we show that vinpocetine reduced superoxide anion production and *gp91^phox^* mRNA expression and restored total antioxidant defense (as observed by normalized levels of the paw skin tissue to reduce iron and scavenge the cationic radical ABTS, and *Nrf2/Ho-1* mRNA expression). Interestingly, cotreatment of morphine with an inducer of HO-1 results in the potentiation of analgesia, indicating that the stimulating Nrf2/HO-1 signaling pathway induces analgesia, in addition to the widely known antioxidant properties [47, 48]. Our data corroborate other reports that show that vinpocetine reduces oxidative stress in different models, such as carrageenan- and LPS-induced inflammation [24, 26], diclofenac-induced kidney injury [20], and hepatic ischemia-reperfusion [49]. Vinpocetine has also been found to increase the antioxidant activity in the brain, indicating a neuroprotective effect [50, 51]. The antioxidant mechanism of vinpocetine is related to its ability to scavenge a singlet oxygen, superoxide anion, and hydroxyl radical [49, 52]. Therefore, these direct and indirect mechanisms are important for the analgesic effect of vinpocetine.

NSAIDs act by inhibiting the enzymatic activity of COX-1 and/or COX-2 resulting in reduced PGE_2_ formation, which is the reason why NSAIDs have been extensively used as analgesics [22]. PGE_2_ sensitizes nociceptor sensory neurons causing hyperalgesia [12, 38, 39]. In spite of being widely used, these drugs must be prescribed with caution due to their nephrotoxic effect [20, 22, 53]. Vinpocetine is a widely used drug in clinics for the treatment of cerebrovascular diseases with no significant side effects and toxicity reported [29, 30, 32]. Of interest, vinpocetine ameliorates diclofenac-induced kidney injury, indicating a safe preclinical profile with protective properties to the kidney [20]. In addition to lipid mediators such as PGE_2_, peptides such as endothelin-1 play an important role in pain. Nociceptor sensory neurons are activated by endothelin-1 during inflammation resulting in pain in rats [13] and in humans [14]. Moreover, IL-33 and IL-15, which are proinflammatory cytokines, produce pain by inducing the release of endothelin-1 and PGE_2_ indicating that these are important components of inflammation and pain [54, 55]. In terms of ROS, endothelin-1 increases superoxide anion and PGE_2_ production [56], and in turn, KO_2_ increases *endothelin-1* mRNA expression [11], indicating a loop between these components. Hence, targeting the components of this loop may be beneficial. In fact, a reduction in oxidative stress, pain, and inflammation after treatment with selective (clazosentan and BQ-788) or mixed (bosentan) antagonists of the endothelin-1 receptor has been observed [11, 35]. Therefore, the decrease in both *Cox-2* and *endothelin-1* mRNA expression might have contributed to the analgesic and anti-inflammatory effect of vinpocetine.

Targeting cytokines is recognized as an effective analgesic approach [40, 57]. The injection of cytokines such as IL-33, IL-1*β*, and TNF-*α* induces pain or potentiates hyperalgesia in models of acute [58, 59] and chronic pain [54, 60]. Moreover, these same cytokines also contribute to neutrophil recruitment toward the tissue, which increases the inflammatory process by producing more proinflammatory cytokines, ROS, endothelin-1, and PGE_2_ [61–63]. Of interest, not only cytokines but ROS also mediate neutrophil recruitment [64] by regulating actin dynamics in these cells [65]. These cytokines are produced in an NF-*κ*B-dependent manner, which indicates that targeting this signaling pathway can reduce inflammation. Vinpocetine has been shown to target NF-*κ*B in different inflammatory disease models [20, 24, 25, 27, 33, 66, 67] and also in human cells [68]. This effect in human cells was observed by the reduced levels of phosphorylation and degradation of I*κ*B*α* in the vinpocetine-treated peripheral blood mononuclear cells (PBMC) from patients with acute ischemic stroke [68]. In the present work, we showed increased levels of I*κ*B*α* (the cytoplasmatic inhibitor of NF-*κ*B) upon treatment with vinpocetine. Therefore, diminishing NF-*κ*B activation explains the reduced cytokine production. The mechanisms by which vinpocetine can reduce NF-*κ*B activation are related to directly targeting IKK [25] or inhibiting the phosphorylation of the upstream enzyme Akt [27]. ROS, including the superoxide anion, also activate the NF-*κ*B signaling pathway [9, 16, 69, 70]; therefore, the antioxidant activity of vinpocetine may also account for the inhibition of NF-*κ*B activation and the inhibition of downstream cytokines reported here. In summary, vinpocetine may reduce NF-*κ*B activation by targeting upstream enzymes [25, 27] or by inhibiting oxidative stress [9, 16, 69, 70].

## 5. Conclusion

Vinpocetine reduced inflammatory pain in a mouse model with a direct role of oxidative stress in the genesis of pain, which was triggered by KO_2_. The vinpocetine analgesia involved diminished the recruitment of innate immune cells (e.g. macrophages and neutrophils) and diminished tissue oxidative reactions (e.g. normalization of the ability of endogenous tissue to reduce iron and scavenge ABTS and *Nrf2/Ho-1* mRNA expression and reduction in the superoxide anion system (NBT assay and *gp91^phox^* mRNA expression)). Vinpocetine also reduced the production of proinflammatory and prohyperalgesic cytokines of the IL-1 family (e.g., IL-33 and IL-1*β*) and the tumor necrosis factor family (e.g., TNF-*α*). This effect is possibly related to the ability of vinpocetine to inhibit NF-*κ*B activation as observed by an increase in the protein levels of I*κ*B*α*. Our data highlight the wide applicability of vinpocetine in models of pain, suggesting that this drug is an attractive alternative for the treatment of inflammation and pain. Thus, we expanded the analgesic properties of vinpocetine in a mouse model with a direct role of ROS in triggering pain and inflammation.

## Data Availability

The data used to support the findings of this study are available from the corresponding author upon request.
